# Brazilian portuguese validation of the patient-reported outcome measure for urethral stricture surgery (USS-PROM) questionnaire

**DOI:** 10.1590/S1677-5538.IBJU.2023.0602

**Published:** 2024-04-10

**Authors:** Karolina Brochado Jorge, Gabriela Silveira Viana, Renan Trevisan Jost, Eduardo Brasil Rabolini, Renan Timoteo de Oliveira, Antonio Rebello Horta Gorgen, Patric Machado Tavares, Tiago Elias Rosito

**Affiliations:** 1 Universidade Federal do Rio Grande do Sul Porto Alegre RS Brasil Programa de Pós-Graduação em Ciências da Saúde: Ginecologia e Obstetrícia, Universidade Federal do Rio Grande do Sul, Porto Alegre, RS, Brasil;; 2 Hospital de Clínicas de Porto Alegre Divisão de Urologia Grupo de Urologia Reconstrutiva e Infantil Porto Alegre RS Brasil Grupo de Urologia Reconstrutiva e Infantil, Divisão de Urologia, Hospital de Clínicas de Porto Alegre, Porto Alegre, RS, Brasil

**Keywords:** Urethral Stricture, Patient Satisfaction, Quality of Life

## Abstract

**Introduction:**

Urethral stricture is a common, albeit complex, condition that predominantly affects men. The aim of this study was to translate, culturally adapt, and validate the Patient-Reported Outcome Measure questionnaire for patients undergoing urethroplasty (USS-PROM) into Brazilian Portuguese using validated psychometric criteria.

**Materials and Methods:**

The process involved translating and culturally adapting the original USS-PROM into Brazilian Portuguese (USS-PROMbr), synthesizing, back-translating, cross-culturally adapting, and analyzing the pre-final version with experts from our committee. This pre-version was administered to 10 patients who had undergone urethroplasty by the Reconstructive Urology team at the Hospital de Clínicas de Porto Alegre for face validation, linguistic, and semantic adjustments, resulting in the final USS-PROMbr version. Subsequently, well-established psychometric criteria, including content validity, internal consistency, and test-retest reproducibility, were assessed after administering the questionnaire to a total of 56 patients, with 50 of them responding to the test and retest.

**Results:**

Evaluation of the pre-final version identified 15 questions as clear, and only one question was considered somewhat unclear necessitating modifications based on patient suggestions and subsequent reassessment by the research team. Psychometric criteria demonstrated good content validity, with a content validity index exceeding 0.80 for all questions; good internal consistency, Cronbach's alpha of 0.77, ranging from 0.70 to 0.78 with the exclusion of any item, and item-total correlations ranging from 0.33 to 0.67. The test-retest intraclass correlation coefficient was 0.74 for the lower urinary tract symptoms construct (Q1-Q6).

**Conclusion:**

The USS-PROMbr demonstrated acceptable cross-cultural adaptation and psychometric properties, making it a valid and useful tool for evaluating patients undergoing urethroplasty.

## INTRODUCTION

Urethral stricture, one of the oldest and most complex pathologies in Urology (1), significantly impairs the quality of life of patients (2). Presently, there is an incidence of up to 0.6% in the overall male population (3), with a higher prevalence of involvement in the anterior urethra (92.2%) (4). Generally, the etiology can be traumatic, iatrogenic (catheterization or instrumentation), inflammatory, infectious, or idiopathic. Idiopathic and iatrogenic causes are the most common in developed countries, while traumatic causes predominate in developing countries(1, 2, 5). The most common urinary symptoms caused by the urethral stricture are obstructive lower urinary tract symptoms, in addition to hematuria, recurrent urinary tract infections, and bladder stones (6). Complementary exams, especially uroflowmetry and retrograde and voiding urethrocystography aid in the diagnosis and follow-up (2, 7) of this condition after surgical treatment, with urethroplasty being the gold standard (6). Therapeutic success aims to restore urinary flow with a good quality of life (QoL) and without the need for further procedures; thus, patients’ perception of postoperative outcomes and their quality of life has become crucial (8).

Questionnaires have been developed to assess patient-reported outcome measure (PROM) to obtain a true understanding of the "therapeutic success" of specific diseases (9). In 2011, Jackson et al. published and validated the first PROM specifically for patients with urethral stricture, the USSPROM - Urethral Stricture Surgery Patient-Reported Outcome Measure. Developed by a group of British surgeons with extensive experience in urethroplasty, it resulted from a series of meetings and discussions about symptoms reported by men who had urethral stricture and had improved after surgery (10, 11). Through well-established psychometric criteria (10, 12) this tool was validated and designed for assessing patient-reported outcome measure after urethral stricture surgery (urethroplasty). The USSPROM has already been translated and validated in several languages: German, Spanish, Italian, Dutch, and Polish, with excellent results in psychometric criteria analysis (7, 9, 12–15), therefore it would be possible to achieve similar results to Brazilian Portuguese as well.

The aim of this study is to translate, cross-culturally adapt, and validate the USSPROM for Brazilian Portuguese (USSPROMbr) through well-described psychometric criteria to create a validated tool that assesses the quality of care provided to Brazilian patients undergoing urethroplasty for urethral stricture treatment. This will also enable the results to be extrapolated and compared in different studies from centers for reconstructive urology worldwide.

## MATERIALS AND METHODS

This study was approved by the Ethics Committee of our Hospital (IRB: 69400323.8.0000.5327) and all patients signed the Informed Consent Form after receiving an explanation about the study.

### Patients

The sample consisted of 66 patients who underwent urethroplasty at the Hospital de Clínicas de Porto Alegre by the Reconstructive Urology group as a treatment for urethral stricture. All patients were male, Brazilian, over 18 years old, with the diagnosis of urethral stricture confirmed by urethrocystography, and had undergone urethroplasty at least 1 month prior. Patients in the postoperative stage of the first phase of two-stage urethroplasty and illiterate patients were excluded. Ten patients were part of the initial phase of the study; they completed the pre-final questionnaire independently. Subsequently, the research team conducted another questionnaire administration, requesting clarity and an explanation of the patient's understanding of each question. Fifty-six other patients completed the final version of USSPROMbr, and 50 of them completed the questionnaire for a second time, with an average interval of 6 weeks between the two administrations.

## USSPROM

The USSPROM (Appendix 1) consists of 16 questions with different domains. The first domain focuses on lower urinary tract symptoms (LUTS) and comprises six questions derived from the International Consultation on Incontinence Male Lower Urinary Tract Symptoms (ICIQ MLUTS) questionnaire (16), with responses ranging from 0 to 4. In this domain, there is also a question about LUTS-specific QoL and Peeling's voiding picture - related to urinary flow (17). Two questions were added to the postoperative questionnaire to assess patient satisfaction after surgery, forming the second domain. The third domain contains five questions evaluating the overall quality of life and patient's health status, taken from the EQ-5D (18), as well as a visual analog scale related to the patient's current health status.

### Psychometric Evaluation

#### A) Translation, Cross-Cultural Adaptation for Brazilian Portuguese - Face and Cross-Cultural Validity

In the qualitative phase, the process of translating and culturally adapting the USSPROM questionnaire into Brazilian Portuguese was conducted following the Guidelines for the Cross-Cultural Adaptation Process of Self-Report Measure (19). Initially, the original British version of the USSPROM questionnaire was translated by two native Brazilians fluent in English, one non-medical (T1) and one urologist (T2). From these two versions, a single version (T12) was developed after changes and corrections made by the research committee, composed of urologists, general surgeons, a pediatric urologist surgeon, and a psychologist. This version underwent back translation by two native English speakers, non-medical professionals fluent in Brazilian Portuguese (BT1 and BT2), to clarify possible inconsistencies between the original version and the Portuguese translation and to identify conceptual errors, resulting in the preliminary or pre-final version in Brazilian Portuguese. The preliminary version was completed by ten patients. Initially, patients completed the questionnaire individually, and approximately one hour later, it was administered by a medical researcher to assess the understanding of each item and clarify any need for changes to ensure that the patient's comprehension aligns with the intention of the original question. At this point, each patient was asked to evaluate each question on a scale of 1 to 10 regarding clarity/understanding (20). Questions with average ratings of 1 to 4 were considered confusing, those with scores of 5 to 7 were deemed unclear, and those with scores of 8 to 10 were considered clear. The collected data are presented as the mean and standard deviation of the clarity index. Questions considered confusing or unclear were revised. These 10 patients were excluded from subsequent analysis of the final version. Cross-cultural adaptation aims to ensure consistency in content and face validity (12) between the original questionnaire and the new version, according to Beaton et al. (19).

#### B) Content Validity

Content validity, regarding the ability of an instrument's items to represent the construct they are measuring, was assessed in two ways. Firstly, through the results of discussions among the expert committee, consisting of 9 participants, in addition to the discussions with the study translators. Secondly, the expert committee scored each item of the final questionnaire from 1 to 4 (1 = not relevant/irrelevant; 2 = somewhat relevant; 3 = quite relevant; 4 = very relevant) through the Content Validity Index (CVI). The CVI is the sum of responses 3 and 4 divided by the total number of responses for each item. The expected CVI is at least 0.8 and preferably above 0.9 for agreement among experts (21, 22).

#### C) Internal Consistency

Internal consistency aids in the reliability of the instrument, along with reproducibility. It demonstrates whether all sub-items of a construct measure the same characteristic and can consequently summarize the item scores (23). For the analysis of internal consistency, Cronbach's alpha test is used - for both the scale and scale with any one item deleted. Internal consistency and item-total correlation were employed to assess the interrelation between questions within the lower urinary tract symptoms (Q1-Q6) construct; Q1-Q8, which included questions regarding the LUTS-related quality of life and Peeling's voiding picture; Q1-Q9, which included postoperative satisfaction; as well as the quality of life construct taken from the EQ-5D (Q11-Q15). Alpha values equal to or greater than 0.70 are acceptable, as well as item-total correlation above 0.20 (10).

#### D) Reproducibility (test-retest)

Reproducibility is the degree of stability of responses over time. Test-retest reproducibility was assessed by the test-retest, with a mean interval of 6 weeks between applications. The instrument's reproducibility in the test and retest was assessed through the intraclass correlation coefficient (ICC - absolute agreement) and the Spearman coefficient for LUTS questions (Q1-Q6), quality of life (Q11-Q15), and item Q16 - the ruler measuring current health status. A paired t-test comparison was also performed for questions 1-6 related to the lower urinary tract construct and for questions 11-15 related to the quality of life.

## Statistical Analysis

The collected data were presented as absolute and relative frequency (categorical variables), mean, standard deviation (SD), minimum value, and maximum value (continuous variables). All analyses were performed using IBM SPSS Statistics version 26.0, and the adopted significance level was p <0.05.

## RESULTS

A total of 56 men were evaluated, with a mean age of 60.1±15.3 years. Approximately 43% of the participants had completed primary education, 37.5% had incomplete or complete secondary education, and 16.1% had completed higher education. The majority of participants had bulbar urethral stricture (51.8%) or penile stricture (28.6%). The size of the stricture varied between 0.2 and 9.0 cm (mean of 2.5±2.2 cm).

The average time between surgery and the first evaluation was 21.0±21.0 months (1 – 108 months). Table-1 presents the descriptive characteristics of the participants in the final phase of this study.

**Table 1:** Socio-demographic and clinical characteristics of the study group.

		Mean (SD)	Min - Max	Median (IQR)
**Age**, in years (n=56)		60.1 (15.3)	23 - 84	62.5 (50.8-73.5)
**Stricture size**, in cm (n=29)		2.5 (2.2)	0.2 - 9,0	2.0 (1.0-4.0)
**Time from surgery to 1st evaluation,** in months (n=56)		21.0 (21.0)	1 - 108	13.0 (4.3-36.5)
**Time from 1st to 2nd evaluation**, in weeks (n=50)		6.2 (2.7)	1 - 17	5.0 (4.8-7.0)
		n	%	
**Education** (n=54)				
	Up to completed Elementary School	24	42.8%	
	Incomplete or completed High School	21	37.5%	
	Completed Higher Education	9	16.1%	
**Stricture location** (n=56)				
	Bulbar	29	51.8%	
	Penile	16	28.6%	
	Navicular	7	12.5%	
	More than one location	4	7.2%	

SD = standard deviation; IQR = interquartile range

### Instrument Reliability

The evaluation of face validity and clarity and understanding yielded excellent results after analyzing the responses of 10 patients who self-completed the preliminary version, with average scores above 9.8 for clarity and understanding. Only question 16 was considered somewhat unclear by the patients, with an average score of 7.6. Even patients who claimed to have understood question 16 and scored it above 8 suggested changes in its composition when the questionnaire was administered by the medical researcher. This question underwent the most significant alteration after considering patient feedback and receiving approval in an experts’ committee meeting. The mean and standard deviation regarding the clarity/understanding of each item in the USSPROMbr questionnaire are presented in Table-2.

**Table 2:** Mean and standard deviation of each item regarding the clarity/understanding analysis of the preliminary version of USSPROMbr (n=10).

Item	Mean ± standard deviation
1.	10 ± 0
2.	10 ± 0
3.	10 ± 0
4.	10 ± 0
5.	10 ± 0
6.	9.8 ± 0.6
7.	9.8 ± 0.4
8.	10 ± 0
9.	9.8 ± 0.6
10.	9.9 ± 0.3
11.	9.9 ± 0.3
12.	9.9 ± 0.3
13.	9.9 ± 0.3
14.	9.8 ± 0.4
15.	10 ± 0
16.	7.6 ± 1.6

USS-PROMbr = Urethral Stricture Surgery Patient-Reported Outcome Measure Brazilian.

The USSPROMbr is a questionnaire designed for the assessment of patients over 18 years old, fluent in Brazilian Portuguese, diagnosed with urethral stricture with a plan for treatment through urethroplasty or those who have undergone urethroplasty. Content validity was ensured and established through expert group meetings and the Content Validity Index (IVC). The IVC was calculated for the 16 questions in the questionnaire, resulting in 1.0 for questions 1 to 12, 14, and 16, while questions 13 regarding usual activities and 15 concerning anxiety and depression had an IVC of 0.88, still ensuring good agreement and adequate content validity for all items.

The internal consistency of the translated instrument, assessed through Cronbach's alpha, was α=0.77 for the lower urinary tract construct (items 1-6), α=0.82 for items 1-8, and α=0.83 for items 1-9. These values are considered acceptable (≥0.70), indicating good internal consistency. For the quality of life construct (items 11-15), the alpha was 0.57, with Q15 (anxiety and depression) showing the poorest performance (Alpha if the item is excluded = 0.73) (Table-3).

**Table 3:** Results of reliability analyses and internal consistency for the LUTS and QoL domain and Q16.

	Internal Consistency (Cronbach's α a)	Test-retest reliability (ICC b)	Test (mean, SD)	Retest (mean, SD)	SCC c
**LUTS** (Q1-Q6)	0.77 a	0.74* (95% CI: 0.59 – 0.85)^b^	13.8 (5.3)	13.1 (5.2)	0.75 *
**QOL** (Q11 – Q15)	0.57 a	0.62* (95% CI: 0.41 – 0.76)^b^	7.0 (1.6)	7.2 (1.7)	0.59 *
**Ruler** (Q16)		0.80* (95% CI: 0.67 – 0.88)^b^	75.0 (18.3)	71.7 (19.3)	0.85 *

* p < 0.001

a = Cronbach's alpha;

b = Intraclass Correlation Coefficient;

c = Spearman Correlation Coefficient

LUTS = Lower urinary Tract Symptoms; QoL = Quality of Life; Ruler 16 = Score related to the ruler measuring current health status.

**Figure 1:**
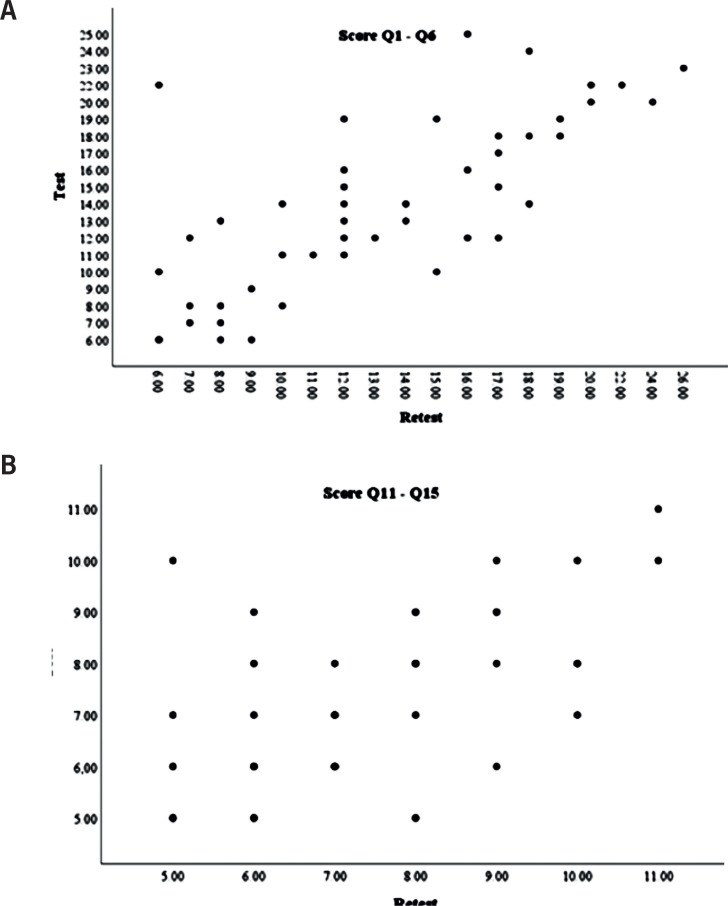
Comparison between scores in test-retest evaluation of LUTS (A) and QoL (B) domain.

Regarding reproducibility (test-retest), the Intraclass Correlation Coefficient (ICC) for the LUTS domain was 0.74; ICC=0.62 for the Q11-Q15, and ICC=0.80 for the ruler Q16 (Table-3). Table-3 also presents the means and standard deviations for the test and retest evaluations and the Spearman correlation test. The Spearman correlation coefficient was 0.75 for the score Q1-Q6 and 0.59 for the score Q11-Q15. In the paired comparison of the sum of scores Q1 to Q6 and Q11-Q15, the results showed no statistically significant difference (p>0.05) between the test and retest evaluations (Figures 1A and 1B). The correlation coefficient between the measurements was greater than 0.50 for all items, except item 14 - the question about pain.

## DISCUSSION

The assessment of surgical outcomes in urethroplasties has undergone a significant shift in recent years, expanding its focus beyond the traditional medical-clinical perspective of restoring urinary flow without the need for additional procedures. Nowadays, it also considers the patients’ perspective regarding therapeutic outcomes (6, 24–26). Since 2011, Jackson et al. enabled a more comprehensive analysis of patients with urethral stricture after creating and validating the USSPROM, the first questionnaire specifically validated for evaluating patients with urethral stricture before and after urethroplasty. The USSPROM results, combined with data from complementary exams such as uroflowmetry, provide a more comprehensive view of the patients’ micturition status, highlighting other lower urinary tract symptoms like hesitation, pain, and urgency, allowing for the comparison of postoperative results among different specialized centers in the field. Result comparisons offer critical insight into the provided care, facilitating the implementation of improvements (7, 15). Despite the importance of the patients’ perspective, there is currently no similarly validated tool in Brazilian Portuguese. Therefore, we validated the Brazilian version of the USSPROM, aiming for a better assessment of our patients. The demonstrated psychometric properties of the Brazilian version (USSPROMbr) are quite appropriate and comparable to the British USSPROM and its translations and adaptations into other languages (7, 10, 12–15, 27), albeit with some differences.

In the literature, although some divergences, there is a consensus to consider validity and reliability as the main measurement properties of instruments. Reliability concerns the stability, internal consistency, and equivalence of a measure (22). The search for semantic and conceptual equivalence of the Brazilian version was guaranteed through face and cross-cultural adaptation and validation (19, 22), involving translation into Brazilian Portuguese, synthesis, back-translation into English, evaluation by the expert committee, in addition to self-administration of the preliminary version of the questionnaire to 10 Brazilian native patients with various levels of education and application by the medical researcher. Patient feedback and clarity/understanding scores were considered. The average clarity score for all items was 9.7, with only one item requiring more significant changes for improvement in understanding. This phase provided good language and concept adaptation, ensuring a final version with good cross-cultural adaptation without losing equivalence with the original tool. Similar to the qualitative analysis conducted by expert meetings, the Content Validity Index (CVI) was adequate for all items, as well as the Persian validation, with scores above 0.8 for all items, being 0.88 for two items that are part of the quality of life domain and 1.0 for all others.

The reliability of the new instrument was also guaranteed by its adequate internal consistency and test-retest reproducibility. The Cronbach's alpha coefficient was α=0.77 for the STUI construct (items 1-6); α=0.82 for items 1-8; and α=0.83 for items 1-9, also demonstrating good consistency with questions related to quality of life related to STUI (Q7), Peeling's voiding picture (Q8), and postoperative satisfaction (Q9). The results are similar to validations in other languages, such as Persian (α=0.80 for questions 1-10), Italian (α=0.79 for Q1-6), Spanish (α=0.70 for Q1-6), and Turkish (α=0.79 for Q1-6) (9, 12, 13, 25). The alpha for questions 11-15 seemed to have been compromised by question 15, related to depression and anxiety, which may be related to other factors not covered in questions 11-14 and not related to urethroplasty surgery. Test-retest reproducibility, assessed by the test-retest, with a mean interval of 6 weeks between applications, also presented results that were adequate and similar to the literature (7, 9, 12–15, 27). The mean interval suggested by Terwee et al. is 1-2 weeks, although it does not define an ideal number. However, there is no standard period when compared to other validations published. German and Polish (7, 14) researchers assessed reproducibility over 3 months, Dutch researchers in 20 days (15), and Spanish researchers in approximately 2 months (13). We chose at least four weeks to facilitate our patients’ access to the outpatient clinic, as many come from the countryside, and a short assessment period could cause logistical issues. Based on clinical practice, we also judged that four weeks would not be sufficient to result in significant clinical changes and would not allow patients to remember their responses from the first application. The sample of at least 50 patients in the test-retest was also appropriate according to the literature and was similar to other publications (7, 10, 12–15, 28).

A limitation of the study may be that the correlation between the LUTS domain and maximum flow rate was not evaluated. Uroflowmetry is only a snapshot of the patient's urinary flow, while the questionnaire aims to assess micturition status and its nuances in the last four weeks. Additionally, the result of uroflowmetry is operator-dependent and influenced by many other factors such as a minimum urinated volume of 150mL, symptoms related to BPH, diabetes, and neurogenic bladder, among others (24). The heterogeneity of results in the assessment of this item by researchers from other countries was also a factor that supported our impression. Polish researchers found a strong statistically significant correlation between Qmax and LUTS in the postoperative assessment, while the Dutch found none in both pre and postoperative evaluations. Adequate urinary flow may be the result of significant micturition effort and may even be associated with other complaints, such as post-micturition dripping and hesitation, thus demonstrating the complementarity of these two tools (29).

Our population, although from a single reference center, presented significant heterogeneity in terms of education level, age, severity, and location of urethral stricture, requiring different types of urethroplasty and reflecting the reality in reconstructive urology reference centers (30). This will likely allow us to extrapolate the study results to the general population. The process of translation, cross-cultural adaptation, and validation of USSPROMbr followed rigorous and well-established criteria in the literature. The results of the study indicate that the Brazilian version of USSPROM has adequate psychometric properties, as well as the original tool and the other versions in other languages. This fact reaffirms the demonstrated versatility of this questionnaire, allowing the assessment of patients with urethral stricture from different countries and cultures worldwide and can now also be used in the follow-up of Brazilian patients, representing an essential tool in the field of Reconstructive Urology.

## CONCLUSIONS

The Brazilian version of USSPROM demonstrated good cross-cultural adaptation, and adequate psychometric properties, and is a valid tool for assessing patient-reported outcomes in individuals undergoing urethroplasty, evaluating their micturition symptoms and quality of life.

## APPENDIX

Thank you for completing this questionnaire. The following questions are designed to measure the effect that urethral strictures have on patients’ lives.

Some questions may look the same but each one is different. Please take time to read and answer each question carefully, and tick the box that best describes your symptoms over the past 4 weeks.

If you currently have a urethral or suprapubic catheter (a catheter through the lower abdomen) please start at page 4.

**USS-PROMbr**

Obrigado por responder este questionário.

As perguntas a seguir têm como objetivo medir o efeito que as estenoses de uretra têm na vida dos pacientes.

Algumas perguntas podem parecer iguais, mas são diferentes. Por favor, leia e responda cada questão cuidadosamente e marque a alternativa que **melhor descreve seus sintomas nas últimas 4 semanas.**

Se você está em uso de uma sonda uretral ou supra-púbica (uma sonda na porção inferior do abdome) neste momento, por favor **comece na página 4.**

1. **Você demora algum tempo para começar a urinar?**
  □ 0. Nunca
  □ 1. Ocasionalmente
  □ 2. Às vezes
  □ 3. Na maioria das vezes
  □ 4. Sempre
2. **Como você diria que é a força do seu jato urinário?**
  □ 0. Normal
  □ 1. Ocasionalmente fraco
  □ 2. Às vezes fraco
  □ 3. Fraco na maioria das vezes
  □ 4. Fraco sempre
3. **Você precisa fazer força para continuar urinando?**
  □ 0. Nunca
  □ 1. Ocasionalmente
  □ 2. Às vezes
  □ 3. Na maioria das vezes
  □ 4. Sempre
4. **Enquanto está urinando, você para e recomeça mais de uma vez?**
  □ 0. Nunca
  □ 1. Ocasionalmente
  □ 2. Às vezes
  □ 3. Na maioria das vezes
  □ 4. Sempre
5. **Com que frequência você sente que sua bexiga não esvaziou adequadamente após urinar?**
  □ 0. Nunca
  □ 1. Ocasionalmente
  □ 2. Às vezes
  □ 3. Na maioria das vezes
  □ 4. Sempre
6. **Com que frequência você sente a calça molhar poucos minutos após ter urinado e se vestido?**
  □ 0. Nunca
  □ 1. Ocasionalmente
  □ 2. Às vezes
  □ 3. Na maioria das vezes
  □ 4. Sempre
7. **De modo geral, seus sintomas urinários interferem na sua vida?**
  □ De maneira alguma
  □ Um pouco
  □ Consideravelmente
  □ Muito
8. **Por favor marque o número que corresponde à força do seu jato urinário no último mês.**
9. **Você está satisfeito com o resultado da sua cirurgia?**
  □ Sim, muito satisfeito
  □ Sim, satisfeito
  □ Não, insatisfeito
  □ Não, muito insatisfeito
10. **Se você está insatisfeito ou muito insatisfeito é porque:**
  □ Os sintomas urinários não melhoraram
  □ Os sintomas urinários melhoraram, mas houve outros problemas
  □ Os sintomas urinários não melhoraram e houve outros problemas também

Ao marcar uma opção nas questões abaixo, por favor assinale qual frase melhor descreve seu estado de saúde atual.

### Mobilidade

□ Eu não tenho problemas para caminhar
□ Eu tenho alguns problemas para caminhar
□ Eu sou acamado

### Auto-cuidado

□ Eu não tenho problemas com auto-cuidado
□ Eu tenho alguns problemas para me lavar ou me vestir
□ Eu não consigo me lavar ou me vestir

### Atividades rotineiras (exemplo trabalho, estudo, tarefas de casa, família ou lazer)

□ Eu não tenho problemas para realizar minhas atividades rotineiras
□ Eu tenho alguns problemas para realizar minhas atividades rotineiras
□ Eu não consigo realizar minhas atividades rotineiras

### Dor/desconforto

□ Eu não tenho dor ou desconforto
□ Eu tenho dor ou desconforto moderados
□ Eu tenho grande dor ou desconforto

### Ansiedade/depressão

□ Eu não sou ansioso ou depressivo
□ Eu sou moderadamente ansioso ou depressivo
□ Eu sou extremamente ansioso ou depressivo

Para ajudar as pessoas a dizerem o quão bom ou ruim é o seu estado de saúde, nós desenhamos uma escala (como um termômetro) na qual o **melhor estado de saúde** que você pode imaginar representa **100** e o **pior estado de saúde** que você pode imaginar representa **0**.

Nós gostaríamos que você indicasse nessa escala o quão bom ou ruim está o seu estado de saúde atualmente, na sua opinião.

Por favor, faça isso assinalando na escala ao lado o ponto que representa o quão bom ou ruim está o seu estado de saúde hoje.
